# Draft genomic sequence of lactic acid bacteria: *Lactiplantibacillus plantarum* LP48, *Enterococcus durans* ED01, and *Bacillus subtilis* BS01 with biotechnological potential

**DOI:** 10.1128/mra.00705-26

**Published:** 2026-09-03

**Authors:** Mauro Daniel García, Mari­a Julia Ruiz, Luis Manuel Medina Canalejo, Analía Inés Etcheverría

**Affiliations:** 1 Department of Animal Health and Preventive Medicine, Veterinary Research Centre (CIVETAN), CONICET-CICPBA, Faculty of Veterinary Sciences, National University of the Centre of the Province of Buenos Aires, Tandil, Buenos Aires, Argentina; 2 Food Science and Technology Department–Competitive Research Unit on Zoonoses and Emerging Diseases of the University of Cordoba, International Agrifood Campus of Excellence (ceiA3), Cordoba, Spain; Fluxus Inc., Sunnyvale, California, USA

**Keywords:** genomic sequence, lactic acid bacteria, *Lactiplantibacillus plantarum*, *Enterococcus durans*, *Bacillus subtilis*, artisanal cheese, probiotic, GenBank

## Abstract

We report draft genome sequences of *Lactiplantibacillus plantarum* LP48, *Enterococcus durans* ED01, and *Bacillus subtilis* BS01 isolated from artisanal goat cheese. Strains were identified by PCR, Sanger sequencing, and Illumina whole-genome sequencing. Assemblies revealed strain-specific features.

## ANNOUNCEMENT

Lactic acid bacteria (LAB) are gram-positive microorganisms extensively used in artisanal and industrial food fermentations due to their metabolic versatility (1), antimicrobial activity, and Generally Recognized as Safe status (2). In addition to their technological relevance, many LAB strains exhibit probiotic and health-promoting properties and antimicrobial activities, making genome sequencing an essential approach for characterizing their functional and biotechnological potential (3–5). Here, we report the draft genome sequences of *Lactiplantibacillus plantarum* LP48, *Enterococcus durans* ED01, and *Bacillus subtilis* BS01 isolated from artisanal goat cheese.

Cheese samples from artisanal goat milk production systems in Spain, including Extremadura and Navarra, were processed as previously described (6). Samples (10 g) were homogenized in peptone water, serially diluted, plated on De Man, Rogosa, and Sharpe agar (MRS), and incubated anaerobically at 37°C for 48 h or aerobically at 30°C for 72 h. Presumptive LAB colonies showing circular, smooth, white-to-cream morphology were purified, propagated in MRS broth, and characterized by catalase testing, Gram staining, and cell morphology.

LAB were activated under the aforementioned culture conditions. Genomic DNA was extracted using the Wizard Genomic DNA Purification Kit for gram-positive bacteria (Promega). Species identification was performed by PCR amplification and Sanger sequencing of the 16S ribosomal RNA (rRNA) gene using primers 16SFw (5′-AGAGTTTGATCCTGGCTCAG-3′) and 16SRv (5′-GAAAGGAGGTGATCCAGCCG-3′). Amplicons were purified, bidirectionally sequenced, assembled with DNAstar MegAlign, and compared against GenBank using BLAST (access number: *L. plantarum*: NR_117813.1, *Leuconostoc mesenteroides:* NR_074057.1, *Lactococcus lactis Hordniae*: NR_040956.1). The isolates were initially identified as *L. plantarum*, *L. mesenteroides*, and *L. lactis* subsp. *Hordniae* (6); the latter two, after National Center for Biotechnology Information (NCBI) GenBank quality control and average nucleotide identity (ANI) testing, were subsequently reidentified as *Enterococcus durans* and *Bacillus subtilis*, respectively.

Whole-genome sequencing was performed by MicrobesNG (United Kingdom) through the GetGenome initiative. Strains were grown on MRS medium for *L. plantarum* and on M17 medium for *E. durans* and *B. subtilis*. Genomic DNA was extracted using the DNeasy Blood and Tissue Kit (Promega, USA), and DNA quality was assessed internally by MicrobesNG using Qubit. Sequencing libraries were prepared with the Nextera XT Library Preparation Kit (Illumina) using double DNA input and a PCR extension time of 45 s. Paired-end sequencing (250 bp) was carried out on an Illumina NovaSeq 6000 platform, yielding approximately 30× genome coverage.

Raw sequencing reads were assessed for quality using FastQC v0.12.1 on the Galaxy platform, as described in reference 7, trimmed with Trimmomatic v0.30 using a sliding-window quality cutoff of Q15 (8). From this analysis, we obtained for LP48 two sets of raw reads (1,056,622 sequences; 260.5 Mbp), ED01 raw reads (651,019 sequences; 160.3 Mbp), and BS01 raw reads (824,208 sequences; 203.2 Mbp). *De novo* genome assembly was performed with SPAdes v3.7 (9), and genome annotation was carried out using Prokka v1.11 (8). Default parameters were used unless otherwise stated. Assembly quality was determined using Quast v5.3.0+galaxy1 (10). These data sets were uploaded to Figshare under DOI https://doi.org/10.6084/m9.figshare.33029324. Both Sequence Read Archive (SRA) and assemblies were submitted to GenBank NCBI under BioProject code PRJNA1354809. After GenBank quality control and ANI testing, samples were reidentified as *E. durans* ED01 and *B. subtilis* BS01. Samples were re-annotated with the NCBI Prokaryotic Genome Annotation Pipeline (PGAP) revision 6.10. The draft genomes of the LAB are detailed in Table 1.

**TABLE 1:** Genome assembly statistics of LAB strains isolated from artisanal goat cheese obtained from the NCBI PGAP annotation

Strain	LP48	BS01	ED01
Species	*Lactiplantibacillus plantarum*	*Bacillus subtilis*	*Enterococcus durans*
Genome size (Mb)	3.4	4.2	2.9
GC content (%)	44.5	43.5	38
No. of contigs	33	107	111
Largest contig	816,388	251,640	297,967
Scaffold N50 (Kb)	565.3	101.7	77.3
Scaffold L50	3	13	12
Coding sequence (total)	3,145	4,415	2,794
rRNA	1, 1 (16S, 23S)	3, 3, 4 (5S, 16S, 23S)	1, 2, 2 (5S, 16S, 23S)
Transfer RNA	65	73	55
Coverage	~30×	~30×	~30×
Assembly code	ASM5652238v1	ASM5662936v1	ASM5662935v1
SRA codes	SRS26946111	SRS26946112	SRX30950808
BioSample	SAMN52956092	SAMN52956093	SAMN52956094

These draft genome sequences expand available genomic resources for LAB isolated from artisanal dairy products and provide a foundation for future studies addressing their technological and functional attributes.

## Data Availability

Genome sequence data are available in GenBank under BioProject ID PRJNA1354809 (Submission ID 1354809). BioSample Accession numbers are *L. plantarum* LP48 SAMN52956092, *B. subtilis* BS01 SAMN52956093, *E. durans* ED01 SAMN52956094.
